# Phosphide of Zinc

**Published:** 1876-10

**Authors:** 


### PHOSPHIDE OF ZINC.

Phosphide of zinc is one of the very few articles that have stood the test of experience out of the very many additions to the Materia Medica introduced in recent times. In fact, the large range of nervous affections that resist almost all forms of medication, renders it very important to the practitioner that those remedial agents which have stood the test of experience in treating these often too stubborn affections, be brought prominently before his attention.

The phosphide of zinc, so far, has proven a most efficient agent in the successful treatment of the major part of a certain class of affections. In very many instances it has been far more curative in these cases than phosphorus. Considered in the light of a curative agent, the phosphide of zinc stands alone, not only for the certainty, but for the rapidity of its action, as a nervous tonic and stimulant. Its value, in these respects, has of late been fairly tested in the last and exhausting stages of typhoid and other fevers where the nervous energies have been so far prostrated as to render convalescence, if not doubtful, at least tedious and protracted.

The great therapeutic value of the phosphide of zinc is declared in the most emphatic manner when used in the treatment of that protean form of disease known as *neuralgia*. Compared with phosphorus as a curative agent in neuralgia, the phosphide of zinc has decidedly the advantage in numerous respects. While it is acknowledged by the best observers in the profession, that the former is seldom curative in doses less than one-twentieth of a grain, often calling for as high as one-tenth and one-fourth, the phosphide of zinc yields as reliable and more speedy results in doses of one-tenth to one-eighth of a grain. But few stomachs can tolerate more than one-thirtieth of a grain of phosphorus before manifesting symptoms of irritation, which, in connection with the "matchy" taste soon evolved in eructations following an efficient dose of phosphorus, seldom fails to engender disgust to its farther continuance. Nor are these disagreeable results altogether abolished by any of the multitudinous formulæ now in vogue. These brawbacks and inconveniences are, no doubt, caused by the length of time phosphorus remains in the stomach before it is absorbed. On the other hand, experience with the phosphide of zinc has proven that it enters the circulation far more rapidly than the element, and when administered in doses of one-eighth to one twelfth of a grain, produces its curative influence far more readily and is equally as permanent in therapeutic power.

In neuralgias, especially those that are due to loss of nerve force or exhaustion of the general system from causes that have lowered the constitutional resistance of the vital economy, it acts sometimes so like a charm, as to challenge the gratitude of the patient and the admiration of the prescriber.

Not many years ago, I was treating a case of neuralgia of the fifth pair, which showed so rebellious a front, as to resist such remedies as ergot, belladonna, the bromides, quinine and hyoscyamus, combined with morphia, iron, etc., I resorted to the phosphorous pill, but soon found that the patient could not take enough of the remedy to do any good, before disagreeable symptoms in the stomach presented themselves, which, with he disgusting matchy eructation, engendered such disgust as to cause me to withhold its further use. At this juncture I ordered phosphide of zinc, one-eighth grain every four hours in pill form. After the fourth dose, the pain was materially modified, and in three days there was entire cessation of pain from which date recovery was permanent.

In a case of undoubted angina, I found the phosphide of zinc a most remarkable remedy for good.

I am unable to say what the value of the remedy would be in mania. However, it seems from what I have heard and read of the results obtained from its use in this class of diseases, that it promises to be valuable.

Loss of memory, and impotency, are very favorably influenced by the phosphide of zinc. A gentleman engaged in large mercantile transactions, whose mind was kept intensely occupied with business during the day, complained to me that he found his memory (that had always, up to a few motnhs before, been remarkably retentive), becoming treacherous, that he was getting very forgetful. I gave him two dozen phosphide of zinc pills, requiring him to take one three times a day. I saw him a week after, when he said he saw no difference in his condition. The pills were coutinued three weeks longer, by taking four a day, at the end of which time he was feeling much improved. With this he was encouraged to continue the treatment three months steadily, taking one-eighth of a grain three times a day, improving steadily until he regarded himsclf cured.

Another instance of a loss of sleep from continued mental anxiety, in which the patient complained of being unable to sleep longer than one or two hours during the night. Phosphorus in this case was ill borne. Phosphide of zinc in one-twelfth grain dose every four hours was prescribed. The remedy exercised good control over the case in a few days, which, after six weeks' constant use, restored the lost balance of the nervous system.

The power of the phosphide of zinc in controlling nervous affections that have their orign in exhaustion or depression of the nervous forces, is now beyond a reasonable doubt. When properly administered and persistently persevered in, its curative value in these classes of cases is sufficient to merit the attention and confidence of the profession at large. Having none of the disagreeable effects of phosphorus, its rapid absorbtion into the circulation, its mild stimulant and tonic influences on the nervous centers, recommend it as a valuable curative agent in all forms of disease requiring phosphorus for their cure.

The formula recommended by Prof. Wm. A. Hammond—

℞. Zinci phosphidi, grs. $\frac{1}{01}$.

Ext. nucis vomicæ, grs. $\frac{1}{4}$.

In pills, I have obtained remarkably good results from.—*St. Louis Med. & Surg. Journal.*

